# Combined Aortic and Mitral Disease in Rheumatic Children

**Published:** 1905-11-11

**Authors:** 


### COMBINED AORTIC AND MITRAL DISEASE IN RHEUMATIC CHILDREN.

Dr. F. J. Poynton draws attention to a group of cases in which the mitral and aortic valves are both affected with rheumatic endocarditis. He contends that the usual order of events in these instances is that the mitral valve is first attacked, and later the aortic, sometimes in the same, sometimes in a later rheumatic manifestation. Such a combination of valvular disease is of great importance in actual practice. It indicates, in the first place, the extreme severity of the endocardial inflammation, and hence it is not surprising to find that it is often accompanied by many other rheumatic phenomena; another proof of the severity of the rheumatic process in these cases is the high percentage of deaths. In a series of twenty-one deaths Dr. Poynton found that ten had died before reaching the age of ten years. The patients rarely die from the mechanical difficulties of an injured heart, but almost invariably with the symptoms of another attack of rheumatism.

